# An Inexpensive and Easy to Use Cervical Range of Motion Measurement Solution Using Inertial Sensors

**DOI:** 10.3390/s18082582

**Published:** 2018-08-07

**Authors:** Rafael Raya, Rodrigo Garcia-Carmona, Cristina Sanchez, Eloy Urendes, Oscar Ramirez, Alvaro Martin, Abraham Otero

**Affiliations:** 1 Department of Information Systems Engineering, University San Pablo CEU, Bodilla del Monte, 28668 Madrid, Spain; rodrigo.garciacarmona@ceu.es (R.G.-C.); cristina.sanchezlopezpablo@ceu.es (C.S.); eloyjose.urendesjimenez@ceu.es (E.U.); a.martin141@usp.ceu.es (A.M.); Aotero@ceu.es (A.O.); 2 Werium Solutions, Arganda del Rey, 28500 Madrid, Spain; oscar.ramirez@weriumsolutions.com

**Keywords:** CROM, inertial sensor, wearable sensor, neck pain, diagnosis, assessment

## Abstract

Neck injuries and the related pain have a high prevalence and represent an important health problem. To properly diagnose and treat them, practitioners need an accurate system for measuring Cervical Range Of Motion (CROM). This article describes the development and validation of an inexpensive, small (4 cm × 4 cm × 8 cm), light (< 200 g) and easy to use solution for measuring CROM using wearable inertial sensors. The proposed solution has been designed with the clinical practice in mind, after consulting with practitioners. It is composed of: (a) two wearable wireless MEMS-based inertial devices, (b) a recording and report generation software application and (c) a measurement protocol for assessing CROM. The solution provides accurate (none of our results is outside the ROM ranges when compared with previously published results based on an optical tracking device) and reliable measurements (ICC = 0.93 for interrater reliability when compared with an optical tracking device and ICC > 0.90 for test-retest reliability), surpassing the popular CROM instrument’s capabilities and precision. It also fulfills the needs for clinical practice attending to effectiveness, efficiency (4 min from setup to final report) and user’s satisfaction (as reported by practitioners). The solution has been certified for mass-production and use in medical environments.

## 1. Introduction

### 1.1. The Problem of Spinal Disorders and Neck Pain

Spinal disorders, and neck pain in particular, have a high prevalence and a huge impact on the daily life of people that suffer from it. A recent study reported a global point prevalence (people who suffer the condition in a year) of 4.9% [1], and neck pain has been considered one of the main causes of disability by the Global Burden of Disease Study [2]. Some authors reported a prevalence between 10.4% and 21.3%, with special incidence in computer workers [3]. In some cases, the number rises to 30% [4]. This pathology has a higher incidence in women and the prevalence increases with age. Risk factors for neck pain include genetics and poor psychological health, among others [5].

One of the main causes of neck pain is cervical whiplash, an injury caused by a sudden acceleration-deceleration event. In 2000, there were 901,442 emergency visits for this cause in the United States and 28% of motor vehicle accidents led to neck sprain or strain, the predominant injury under these circumstances [6]. Although in most cases, neck pain can be suppressed, nearly 50% of the affected individuals continue to experience some degree of pain.

Neck pain represents a significant public health problem, incurring substantial social and economic costs worldwide. Moreover, most of the healthcare costs due to musculoskeletal conditions come from neck and back pain [7,8]. In 2005, spine disorders were the third largest source of medical expenses in the United States, after heart disease and stroke [9]. A Canadian study (2004) showed that neck pain represents 14.6% of all musculoskeletal problems reported annually in that country [10]. According to data published by the World Health Organization, there is a substantial and unmet need for rehabilitation services worldwide [11]. Traumatic movement disorders, like cervical whiplash, tie up most of the labor and assistance resources of private clinics in EU. Around 800,000 EU citizens suffer whiplash injuries annually, and 40,000 of these injuries result in long term pain, with an associated socio-economic impact of approximately 10 billion € per year [12].

Neck pain is a prevalent and serious health issue that needs to be tackled head on. Doing so has the potential to improve living conditions for many people and to reduce healthcare expenses. More specifically, advances can be made in two areas: the diagnosis of the neck pain, and its treatment.

#### 1.1.1. Diagnosis of Neck Pain

Since there is a lack of homogeneity in the neck pain prevalence data, it is difficult to measure with precision the impact of this pathology. This is due to errors in neck pain diagnosis, a critical problem that not only affects the initial detection, but also prevents the specialists from understanding the prevalence, predictors, treatment efficiency and clinical course of neck pain. Further research in all these aspects must be conducted [1], and a proper objective diagnosis can help all of them.

Whiplash injuries are often difficult to diagnose due to the lack of objectivity in the exploratory data (which depends on muscle tone and mobility limitations) and the reliance on the patients’ pain sensations, which are not susceptible to medical verification. There are some objective measures, like cervical range of motion (CROM), surface electromyography (EMG) and pressure pain thresholds, that can help with the diagnosis of the pathology and the evolution of therapies and treatments. Several authors state that CROM is a reliable method for diagnosis, showing different values for healthy subjects and patients [13], and confirm that CROM is reduced in all primary movements in patients with whiplash disorder [14].

**Figure 1:**
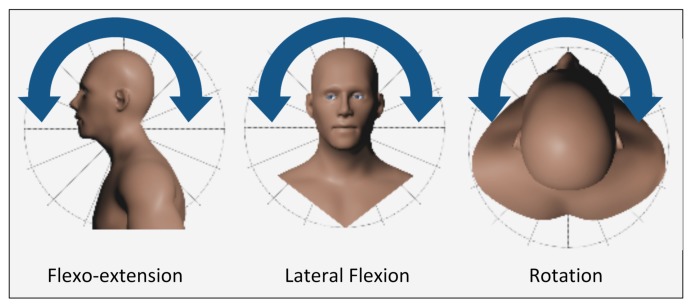
Cervical motions.

Taking these sources into account, we can conclude that the measurement of the patients’ CROM is a good indicator of both the existence of a neck pain pathology and the efficiency of a particular treatment or rehabilitation process. CROM is measured across 3 degrees of freedom and is usually expressed as three pairs of movements: flexion-extension (sagittal plane), left and right lateral flexion (frontal plane), and left and right rotation (transverse plane). These can be seen in Figure 1.

#### 1.1.2. Treatment of Neck Pain

The most common treatment for neck pain is manual therapy, which includes manipulation and mobilization [15], combined with exercise and active movement [4]. There are guidelines that provide a set of exercises for neck disorders [16,17]. These highlight the importance of strengthening the neck through rehabilitation and suggest focusing on this area for treatment. However, the authors conclude that there is a need for research that helps to define the optimal frequency and intensity of these exercises. Such research efforts would need an accurate and, equally important, frequent measurement of the patients’ CROM to evaluate their progress and execute the exercises themselves.

Lluch et al. [18] have studied the effects of passive mobilization (performed by the healthcare professional) and active movements (performed by the patient) in the rehabilitation of patients with chronic neck pain. They were divided into two groups and, for both of them, CROM, surface EMG and pressure pain threshold were measured. The authors conclude that both groups obtained benefits from the treatments, but only the patients that performed the active movements saw improvements in their motor function. This work (like others [19,20]) highlights the need of specific active treatments and state that exercise and mobilization programs provide the best option for the treatment of whiplash-associated disorders. Other authors [17] also recommend focusing on active exercises performed by the patient rather than manual therapy. However, to actually implement such treatments in an effective way, the practitioners need an accurate instrument to measure CROM.

### 1.2. Objectives

In this paper, we present a wearable motion capture solution focused on measuring the range of motion of one joint. We have designed this system following a set of rules that we consider key for the usability in a clinical practice context. All of them revolve around our main objective: to provide a complete and inexpensive solution that enables therapists to evaluate the ROM in 5 min or less, from setup to final report. This article focuses on the CROM since, as it has been presented before, it is a high prevalence problem. However, the system can be adapted to measure the range of motion of other joints.

This article is structured as follows: in the *Materials and Methods* we describe how we developed the solution, which includes a set of hardware devices, a software application and a measurement protocol. In the *Results* we explain both how we validated the proposed system and its mass production and certification processes. In the *Discussion* we put the results in context and propose future work. Finally, in the *Conclusions* we comment our main findings.

## 2. Materials and Methods

### 2.1. CROM Measurement

There are several instruments and techniques that can be used for measuring CROM; from purely mechanical devices like goniometers or inclinometers, to more sophisticated solutions (electro-, magneto- and ultrasonography-based). Their reliability and validity have been studied during the last decades [21,22,23,24,25]. The validity of the instruments discussed in this section is usually tested against X-ray scans and optical tracking devices [26,27], the gold standard in CROM tracking. These are the most accurate solutions, but are not feasible for day to day practice; the repeated used of the former can damage the patient, while the latter is too expensive and too complex to setup. Many clinics own these devices, but do not use them in day to day treatment.

As of today, the manual goniometer is still the most popular tool to measure joints’ range of motion. This instrument is tedious and slow to use, and its accuracy is heavily dependent on the examiner’s experience. Inclinometers suffer from the same limitations.

Another popular device is the CROM instrument (In this article, when referring to the device we will always use the term “CROM instrument”, to distinguish it from the CROM (cervical range of motion) itself.). The CROM instrument is a purely mechanical device, that uses traditional inclinometers and a magnetic reference. This instrument presents some data recording issues, since the patient must maintain a static posture while the practitioner manually writes down the measured angle, which could be displayed in an position inconvenient for taking the measurement. In spite of that, many studies [24,25,27,28,29] have demonstrated that the CROM instrument’s reliability (between tests and testers with the same instrument and subject) and validity are enough for medical practice, being both superior to those of the traditional goniometer. The reported ICCs (Intraclass Correlation Coefficients) fall between 0.82 and 0.98, and standard errors across the 3 movements pairs between 1.6${}^{\circ }$ to 2.8${}^{\circ }$ [26,30]. However, these studies differ in the normal ranges measured by the CROM instrument, ranging from 59.1${}^{\circ }$ ± 18.2${}^{\circ }$ [24,28] to 80.2${}^{\circ }$ ± 13.4${}^{\circ }$ [27] for the same movement (extension). One study in particular [27] compares the validity of the CROM instrument against an optoelectronic system, showing low measurements (50.4${}^{\circ }$ ± 14.4${}^{\circ }$) for the CROM instrument, matching the values obtained by the optoelectronic device. This leads us to think that the real values fall near these measurements. Despite these discrepancies and its other limitations, the CROM instrument is probably the most popular device in physiotherapy clinics.

A recent development in the CROM field are IMU (Inertial Measurement Unit) sensors, which integrate a 3D accelerometer, a gyroscope and a magnetometer. These sensors measure angular velocity, acceleration and the Earth magnetic field. Devices based on an IMU can be easily integrated with an information system, therefore capturing ranges of motion digitally and sending them to other devices. Thus, the practitioner does not need to record the values by hand. Moreover, advances in MEMS (Micro-Electro-Mechanical Systems) technology have reduced the size, weight and power consumption of IMUs, making them suitable for use in wearables.

There have been several studies that have tested whether inertial sensors could be used to measure CROM. Prushansky et al. [31] compared the CROM measurements of a digital inclinometry (a MEMS that measures inclination in only two axes) with those of an ultrasound device. As could be expected, the study revealed no significant differences between instruments for flexion-extension and lateral flexion movements, but the same could not be said about rotation. Another study proved that accelerometer-based systems are reliable for measuring the regional breakdown of spinal motion [32]. These works show that a solution for measuring CROM based on inertial devices is not only feasible, but reliable. Iosa et al. [33] state that wearable devices based on inertial sensors are ready for routine clinical practice.

Despite all the solutions available, most physiotherapy clinics do not have precise tools for measuring range of motion. Most practitioners rely on simple instruments (goniometers or inclinometers) whose measurements are not digitally recorded.

### 2.2. Proposed Solution

We have consulted with 40 practitioners as to why the available solutions are not widely used in the clinical practice. They cite high cost, poor usability, long setup time or functionality that does not fit the therapists’ needs as the main barriers for adoption. This is especially egregious with inertial motion capture systems, since they could provide an alternative to the current gold standard in CROM measurement, optical tracking systems, which are not suited to the clinical practice. Some reasons for the rejection of readily-available inertial solutions are complex and lengthy setups, difficult usage or inadequacy of the provided data. This is only natural, since none of the available systems were designed with the practitioners real needs in mind, but to be used in research environments and, even if these solutions can provide useful data, they are, in the practitioners’ opinion, not worth the effort. For instance: there are solutions that provide a complete biomechanical analysis, but all this information is not necessary for the study and treatment of many injuries, which involve just one body joint. We have also found that a common problem is that the instruments deliver data in a format that is not usable for the practitioner.

We used the information extracted from these interviews with healthcare professionals to design a solution especially tailored to the clinical practice, trying to avoid the pitfalls that affected other designs. After the interviews with the therapists and medical doctors, the following design principles emerged:

- Complete, out of the box, solution
- High precision, at least as good as the CROM instruments used in the clinical routine
- Easy to use
- Inexpensive
- Suits the needs of clinical practice (it should require less than 10 min (ideally less than 5 min) of complete test time, from setup to report)
- Produces electronic reports with the information that therapists need (CROM in 3 axes)
- Aggregates information from several subjects and multiple measurements from the same subject

To the best of our knowledge, this set of requirements is not fulfilled by any of the systems available in the market or the literature.

**Figure 2:**
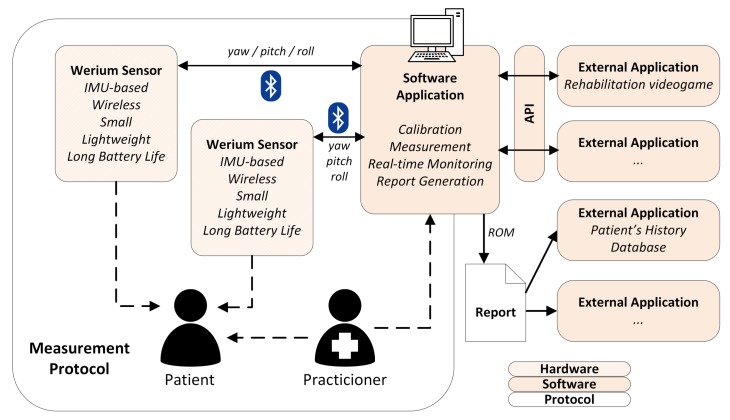
Complete solution overview.

The proposed solution takes advantage of the capabilities of inertial sensors, and approaches the stated design principles from three points of view: hardware sensors, a software application and a measurement protocol. Only through the combination of these three components, which can be seen in Figure 2, our requirements can be achieved.

We aimed to get the best possible CROM measurement while tracking as few points of the patient’s body as possible. Each tracked position needs a separate sensor, and this not only increases the price of the solution, but also burdens the practitioner with placing it, complicating the measurement protocol and increasing the test time and the possibility of human error. The ideal configuration on this regard would be just one sensor, but the literature [34] suggests that a minimum of two sensors is needed, to achieve a higher precision and compensate for unwanted body motions. For instance: subjects could hide a neck problem by compensating the movement with their back. Therefore, the proposed solution uses two hardware sensors. One of them is placed on the mobile segment and the other one is placed on a point of reference. The relative measurement between them offers an accurate angular measurement, compensating the effect of the absolute movement.

These wearable devices are detailed later, but it is important to note that they were designed to be as light (<200 g) and small (4 cm × 4 cm × 8 cm or less) as possible. To simplify the setup, they also need to be wireless and have a long lasting battery (>2 h). To achieve this, the sensors offload the most expensive calculations to an external system: a standard inexpensive computer already available in most clinics (see Figure 2). Therefore, the sensors relay motion data as a combination of yaw, pitch and roll, and the software application combines both sensor’s measurements to produce the subject’s CROM.

To maximize the intertest and intertester reliability, and to reduce the setup time, the calibration process needs to be fast and reliable. This process is directed from a software application, which also allows the practitioner to monitor the subject and that produces the reports. To future-proof the solution and offer other interesting external applications and features (like videogames-based rehabilitation), this application needs to offer a programatic interface (see Figure 2).

Finally, the solution is paired with a clear and quick measurement protocol, an aspect that, in the opinion of the consulted practitioners, is as important as the hardware itself. We shall present each of the three components of the solution (sensors, software applications and protocol) in more detail in the following sections.

### 2.3. Sensors

The sensors development was based on previous work from the authors, focused on head-mounted interfaces in the field of Augmentative and Alternative Communication (AAC) for children with cerebral palsy [35,36]. The concepts introduced in these articles have been instrumental in the development of the device described in this section.

**Figure 3:**
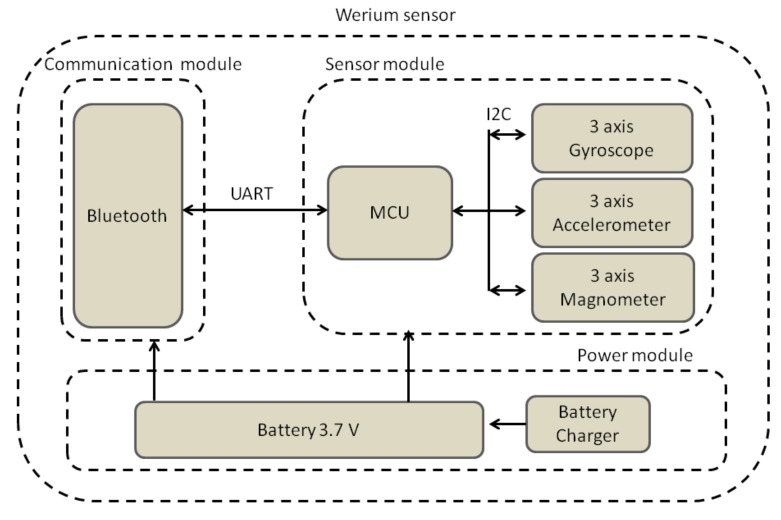
Sensor block diagram.

Each sensor has three main blocks: sensor, communication and power modules. For the pre-industrial device, each of these modules was implemented in a separate printed circuit board (PCB). Their structure can be seen in Figure 3.

The sensor module contains a MEMS-based IMU with 9 degrees of freedom (9-DOF). This IMU integrates a 3-axis accelerometer (ADXL 345 from Analog Devices), which measures acceleration due to gravity and motion, a 3-axis gyroscope (ITG-3200 from Invense) which measures angular velocity, and a 3-axis compass (HCM5883L from Honeywell), which measures Earth’s magnetic field. ADXL 345 has an output resolution measurement of up to ±16 g, a 4 mg/LSB factor scale with 13 bits full resolution for each axis. Its current consumption in measurement mode is 450 µA with typical voltage supply of 3.3 V. ITG-3200 has a full-scale range of ±2000 degrees per second with 16-bit resolution. Its current consumption in measurement mode is 3.2 mA. Finally, HCM5883L sensor has a field resolution of ±4800 µT with 12-bit resolution. Its current consumption in measurement mode is 280 µA when supplied with 3.3 V.

The sensor module also contains a microcontroller unit (MCU) (8-bit AVR, 8 MHz, 32 KBytes of flash memory), responsible for acquiring the sensors data via I2C and computing the angular orientation (yaw, pitch and roll) before forwarding it to the communications module.

The fusion of the signals from a 3D accelerometer, a 3D gyroscope and a 3D magnetometer has been done using a Direction Cosine Matrix (DCM) algorithm [37]. The ROM is estimated using the Euler angles from the Direct Cosine Matrix from the sensor, following Equation (1), where $R_{G}$ is the rotation matrix during calibration (global reference corresponding to the neutral posture). $R_{s}$ is the rotation matrix during the task. These equations are applied for both sensors and the ROM is the result of the substraction of both estimations in order to avoid compensations due to trunk movements.

(1) $$\begin{array}{cc} R_{GS} & =R_{s}\cdot {\left(R_{G}\right)}^{-1}\\ \alpha & =\arctan(-R_{GS}\left(2,3\right)/R_{GS}\left(3,3\right))\\ \beta & =\arcsin(R_{GS}\left(1,3\right))\\ \gamma & =\arctan(-R_{GS}\left(1,2\right)/R_{GS}\left(1,1\right)) \end{array}$$

The communications module contains a Bluetooth radio chip (2.4 GHz, class 2 radio, 20 m range, slave mode and on-chip antenna), connected with the MCU via UART at 57,600 Kbps.

Finally, an USB LiPo Charger and a single LiPo battery (3.7 V, 250 mAh) are integrated in the power module. The battery provides autonomy of up to 3 h. The charger employs a constant-current/ constant-voltage (CC/CV) charging mode (maximum current of 500 mA) and can be charged using an standard mini-USB plug. The device has an on-off switch and two LEDs to indicate the charging status.

**Figure 4:**
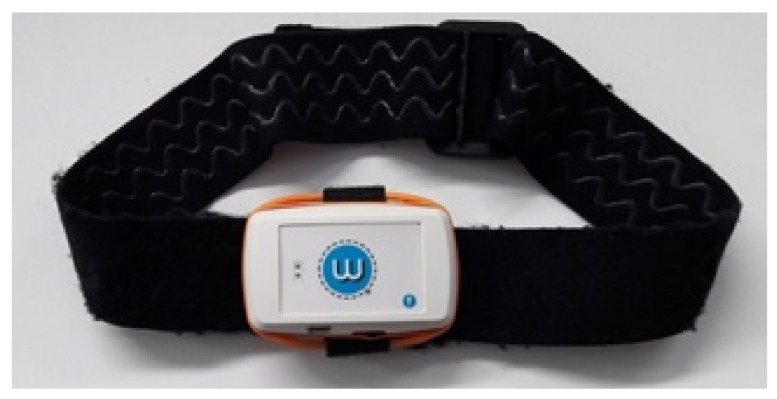
Sensor with the forehead strap.

A commercial ABS enclosure (Ingress Protection (IP) level 41, Underwriters Laboratories (UL) certification 94) is used to encapsulate the modules described above. This housing has 18 mm of external height, 43 mm of external width and 68 mm of external depth, fulfilling our needs for a small-sized and lightweight (200 g) device. The sensor can be placed on the patient’s body in two ways: (a) with a flexible and an adjustable strap, or (b) with adhesive tape. The former was designed to fix the sensor to the patient’s forehead, and the latter for the patient’s back. Figure 4 shows a sensor with its enclosure and the strap.

### 2.4. Software Application

ISO9241-Part9 [38] defines three key specifications for Human-Computer Interfaces usability: effectiveness, efficiency and user’s satisfaction. Effectiveness measures if the system can produce the desired result or fulfill the expected functionality. Efficiency studies the capacity and accuracy for fulfilling the specifications, but in relation to the resources expended. Finally, user’s satisfaction evaluates easy-to-use design, comfort and acceptance. We have taken these three factors into account in order to optimize the software application in clinical practice. Key factors were categorized according to the design principles and requirements.

Effectiveness is related to the numbers of errors the practitioners make when attempting to measure CROM. These errors include unintended actions, mistakes or omissions. To avoid these problem, the application flow must be intuitive, clear and led to an efficient completion.

The software component of the solution was designed to provide several features: CROM measurement, sensor calibration, real-time monitoring and report generation. These were specified by the consulted experts as their biggest needs. We decided to offload further capabilities to external software, in order to minimize the application’s complexity and reduce errors. This is done through an API (Application Programming Interface).

Out of the previously enumerated features, the CROM measurement needs to be optimized the most, since it will see heavy usage during medical practice. Therefore, we have designed the application so the patient’s information (personal data, pathology and so on) is filled during the first session and stored for later use. The remaining time of the first session will be spent taking measurements through the protocol defined later. This means that, for the following sessions, only the measurement process is necessary. Nevertheless, there is an option to add a new pathology for the same body joint or other patient’s information if needed.

Efficiency is measured in terms of task time; how much time the therapist needs to successfully complete an assessment of CROM.

**Figure 5:**
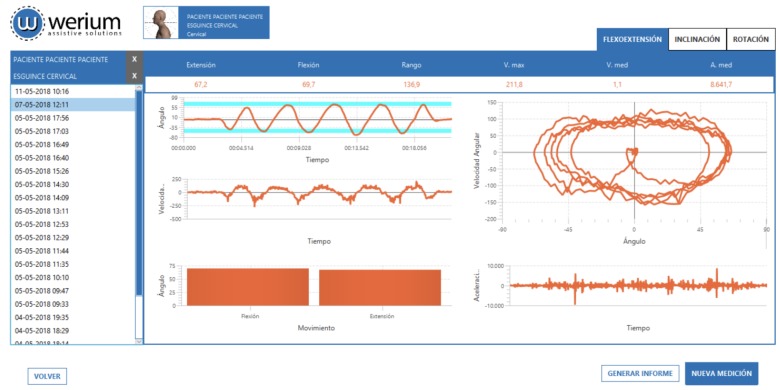
Sample CROM report.

Another heavily requested feature was a way to compare the results of different sessions for the same patient. Therefore, we optimized the application so the practitioner can select different trials for a given subject and export the data as a graphical report. This way, the practitioner will be able to easily examine the patient’s progress. A sample report can be seen in Figure 5.

The application is programmed in reflect varia and it has been fully tested in Windows and Linux. The application requires that the operating system supports Bluetooth 4.0. The sensors only need to be paired with the PC once. Currently the only language it supports is Spanish, since all the practitioners that have tested (and are testing it) are from Spain. However, it is designed to be easily internationalized (replaceable Strings).

### 2.5. Protocol Definition and Sensor Validation

**Figure 6:**
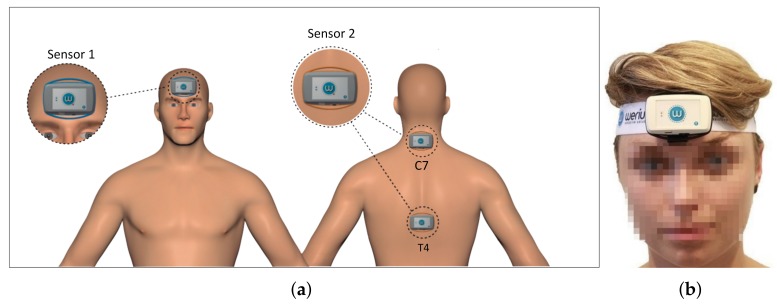
(**a**) Placement diagram; (**b**) Actual placement.

We have defined a protocol for CROM measurement using two sensors and the software application described previously. In this section we explain the protocol and how we have used it to evaluated the sensors performance for three types of movements (flexion-extension, lateral flexion and rotation) with two different placements of the sensors. The first sensor was always located, with the help of a strap, on the forehead of the subject, and the second one was placed, using a small piece of double adhesive tape, over the C7 or T4 vertebrae (chosen following the recommendations of Theobald et al. [34] and of the consulted practitioners), see Figure 6. Consequently, the protocol explained in this section was performed four times for each subject (two repetitions for each of the two sensor placement configurations).

First, the subject is instructed to put on a t-shirt, supplied by the tester (the same person for all the subjects), with a hole in its back (30 cm diameter) so that the inertial sensors can be properly placed. All kind of accessories that could affect the measurement process must be taken off (e.g., hats, jewellery and eyeglasses). Then, the subject is sat down on a chair with their feet flat on the floor, straight back, shoulders relaxed, and the knees making a 90° angle.

Then, the tester plays a video showing the movements that the subject needs to perform. In addition to playing the video, the tester also explains to the subjects other important details, so they can perform the protocol correctly: The subject is told to not separate their back from the chair’s backrest nor touch the sensors while performing the motions; every movement must extend until the subject is limited by tightness or discomfort, but never pain; and the shoulders must not be moved.

Concerning specific motions, during the lateral flexions, the subjects are recommended to take a reference point and use it to avoid rotations. For rotations, in order to avoid compensation movements, the subject was told to refrain from any motion in the other two axes. In this last case, telling the subjects to look at an imaginary horizontal line in front of them reduced this effect.

When the video ends, the tester attaches the two sensors (already switched on and paired with the software application) according to one of the two possible placement configurations. After that, the patient is asked to maintain a neutral position for calibration: nose, mouth and chin vertically aligned; eyes at horizontal level; and ear lobes and the base of the nose also at horizontal level, and the practitioner calibrates the sensors by clicking a button in the software.

After the calibration (0° for every axis in the neutral position), the subject is told to perform the following motions:

1. Three consecutive flexion-extension movements, starting from the neutral position and moving to the maximum flexion position first.
2. Three consecutive lateral flexion movements, starting from the neutral position and moving to the maximum right lateral flexion position first.
3. Three consecutive rotations, starting from the neutral position and moving to the maximum right rotation position first.

During the protocol, the order of the movements was randomized. Using a software-based random number generator, random sequences were generated for each of the three movements. Random sequences were also generated for positioning the sensors first in C4 or in T7. In this way, the possible effects derived from the influence of one movement in the following ones are eliminated.

After a short rest (30–40 s), this protocol is repeated, with the subject performing the three motions again. This is the second set for the same sensor placement (either T7 or C4).

Then the tester places the second sensor in the alternative configuration (C7 if it was on T4, and vice versa), recalibrates the sensors, and asks the subject to repeat the motions twice (two sets). Again, following a random order for the movements.

If the subject did not follow the instructions, the measurement was not considered valid. In these situations, the tester has to explain how the motions should be performed again and repeat the measurements from the beginning.

#### 2.5.1. Participants

A sample of 27 asymptomatic subjects (17 males, 10 females; age (mean ± SD): 26.63 ± 9.44; age range: 18–53 years), with no history of neck pain or condition affecting the cervical spine region in the last year was selected. This population was selected to validate the proposed system against the CROM normal ranges published in the literature.

#### 2.5.2. Measurements

The software application computes the complete CROM of the subject, expressed as angles from the neutral position in the 3 axes, from the motion capture data sampled every 20 ms from each sensor (forehead and C7 or T4). This information is stored in an spreadsheet report.

We have characterized the CROM using two different perspectives: as 3 full-movements (flexion-extension, lateral flexion and rotation), or as 6 half movements (flexion, extension, right lateral flexion, left lateral flexion, right rotation and left rotation). The half movements use the neutral position as reference.

The spreadsheet report also computes the mean of the maximum range of motion for every movement (be they half- or full-). Since each subject performs the motions 3 times per axis for each set of movements, this mean is calculated over the 3 maximum range values for each motion, patient and set.

#### 2.5.3. Data Analysis

We have analyzed the two sets of three motions for each sensor placement. This analysis was performed for the two aforementioned perspectives: considering each motion as one full movement or as two half movements.

To be aware of the central tendency and the dispersion of every motion, we obtained the mean and the standard deviation for each of the two sets of 3 repetitions. This was done separately for each sensor placement (C7 and T4).

To measure the test-retest reliability of the proposed procedure, we calculated the appropriate intraclass correlation coefficient (ICC) and its confidence interval (95% confidence interval (CI) for ICC). In this case, the ICC should reflect the variation in measurements taken by the sensors on the same subject under the same conditions, considering that the tester effect is negligible. Following the experimental design proposed by Koo et al. [39], we chose a two-way mixed effect model (since the type of reliability study is a test-retest one), a multiple measurements-based type and an absolute agreement definition (measurement would be meaningless if there is no agreement between repeated measurements) (ICC was obtained using the icc function of R (v.3.2.3): *icc (ratings, model = c("oneway", "twoway"), type = c("consistency", "agreement"), unit = c("single", "average"), r0 = 0, conf.level = 0.95)*).

We also calculated the difference in every motion between the two sets (for the same placement) as the absolute value of the subtraction. The mean of these differences for each movement was computed and labeled “Mean difference between sets”. Finally, the difference between the mean of each first set of experiments and the corresponding second set was labeled “Difference between means”.

## 3. Results

### 3.1. Sensor Validation Results

Table 1 shows the results for the two sets of motions performed with the second sensor placed in C7, considering half movements (flexion, extension, right lateral flexion, left lateral flexion, right rotation and left rotation). Table 2 shows the same data as Table 1 but with the second sensor placed in T4. Table 3 shows the results for the two sets of motions performed with the second sensor placed in C7, considering full movements (flexion-extension, lateral flexion and rotation). Table 4 shows the same data as Table 3 but with the second sensor placed in T4.

We also assessed the interrater reliability comparing the sensors with an optical tracking system (Vicon). This experiment was carried out by measuring the cervical ranges (the 6 half movements following the previously described protocol) of a sample of eight asymptomatic subjects using both the optical tracking device and the proposed sensors.

For this sample, the suitable ICC and its confidence interval (95% CI for ICC) were obtained. As we wanted to reflect the variation between two raters that measure the same group of subjects, we chose a two-way mixed effect model (because the selected raters are the only raters of interest), a single measurement-based type and an absolute agreement definition to calculate the ICC. We obtained an ICC of 0.93 (95% CI: 0.88–0.96), which shows the accuracy of our solution when compared with the gold standard in biomechanics.

### 3.2. Software Application Usability

For the software component we wanted to evaluate three usability metrics: effectiveness, efficiency and user satisfaction. Effectiveness is very complex to measure objectively, but the *Software Application* section explains how we tried to design the tool as effective as possible.

To measure efficiency, we have performed a pilot test with ICOT, the second biggest network of health centers in Spain, with 33 centers and more than 1200 practitioners. Their therapists specialized in neck pain (different individuals from those consulted for the design, and therefore unfamiliar with the system), were asked to perform CROM assessment session and clock the time taken. The mean value was just under 4 min [40]. This time is much lower than the 10 min suggested by the consulted practitioners to consider the system useful for medical practice.

Regarding effectiveness and user’s satisfaction, ICOT neck pain practitioners in personal interviews described the system’s simplicity and time to perform the assessment as its best characteristics, and they reported that they would like to continue using the system in the clinical routine. At the present time we lack more objective metrics regarding the effectiveness and usability of the system, but we are carrying out a more extensive pilot test within ICOT which will be followed by a questionnaire. Preliminary results of this ongoing second pilot test are promising.

### 3.3. Mass Production and Certification

After the pre-industrial sensor was properly validated, we proceeded with the modifications needed for its mass production and use in medical environments. First of all, to improve the device’s safety, we switched the surfaces that could make contact with the skin (enclosure, strap, adhesive tape) to hypoallergenic materials, to avoid reactions or irritation.

The three modules have been integrated in a single PCB using SMD (surface mounted devices) components, minimizing the size and weight of the device and enabling mass production in an assembly line with RoHS (Restriction of Hazardous Substances) compliance. This way, we can reduce the effects on the sensors precision due to soldering (variable temperature, handling, short circuits, etc.), vibrations or an uncontrolled environments (variable humidity or temperature, dust, etc.).

Concerning wireless communications, the mass-produced version uses a different Bluetooth IC (integrated circuit), in order to comply with EU and USA radio equipment directives (RED) and electromagnetic compatibility (EMC). This is especially important if we consider that the device must operate near medical equipment.

The LiPo battery has been replaced with a coin Lithium cell of similar capacity, paired with a protection circuit. On top of that, the power supply’s status is continuously monitored to improve the electrical safety and remove any possible risk of damage.

Finally, we defined a manufacturing and checking process that allows for quality control, error tracking and regulation compliance. The mass-produced sensor has passed the ECM (electrochemical mitigation) testing (UNE-EN 55022:2011, UNE-EN 55024:2011) and has achieved CE marking for commercialization in EU market.

## 4. Discussion

In this article, we have presented the design of a solution for measuring CROM, based on a pair of wireless wearable inertial sensors, a software application and a measurement protocol. The proposed solution is inexpensive, simple to use and the setup requires little time. In this section, we shall address some aspects of the work that merit some discussion and introduce several ways to further develop the proposed solution.

### 4.1. Reliability

According to Koo et al. [39], ICC values between 0.75 and 0.90 indicate good reliability, and values greater than 0.90 indicate excellent reliability. Our results show good or excellent reliability (with the exception of left lateral flexion with T4 placement, see Table 2) for half movements, and excellent reliability for full movements. It can also be seen that the T4 setup offers better reliability (as suggested by Theobald et al. [34]) than the alternative location at C7. The ICCs obtained in the T4 setup have more narrow confidence intervals than in the C7 setup, indicating that the measures are more accurate. The CIs are even tighter for full movements.

Our results show moderately high standard deviation values, ranging from 6.51° (right lateral flexion, set 1, C7, Table 1) to 14.77° (extension, set 2, T4, Table 2) for half movements and from 13.18° (lateral flexion, set 1, C7, Table 3) to 19.95° (flexion-extension, set 2, T4, Table 4) for full movements. Moreover, it should be noted that the standard deviation is always lower for lateral motions, independently of the sensor placement. These results are similar to other studies [22,23,24]. These high standard deviation values are expected due to factors such as gender or age having a strong influence on the CROM of the individuals.

The difference between means in our results was low, ranging from 0.06° (flexion-extension, T4, Table 4) to 2.27° (flexion-extension, C7, Table 3). The mean difference between sets was also low, with values from 2.85° (left lateral flexion, C7, Table 1) to 6.65° (extension, T4, Table 2). This parameter is particularly stable for all values (specially full movements), confirming the excellent reliability suggested by the high ICC.

When assessing the interrater reliability comparing the sensors with an optical tracking system (Vicon) we obtained an ICC of 0.93 (95% CI: 0.88–0.96). This result shows an excellent interrater reliability of our solution when compared our sensors with the gold standard.

### 4.2. Validity

As discussed in the *introduction*, there is a big heterogeneity in the values found in the literature for the measurement of CROM [24,27,28,41]. This complicates the validation of new devices or measurement protocols. We have decided to compare our results to the values published in the literature [27] for an optoelectronic system, which is considered one of the best instruments for kinematics measurement [42]. The published results for CROM using an optoelectronic system are shown in Table 5. However, it is important to keep in mind that we are using a population that, as could be expected, is different from the one used with the optoelectronic system [27].

To properly compare these values with our results, we need to look only at half movements. In general, our values match those of Table 5 (r>0.95 for all cases, with r= Pearson Correlation Coefficient). Moreover, with the C7 placement, our means for both sets fall within 4° of the ones published for the optoelectronic device for flexion, extension and both lateral flexions. However, the difference is larger for both rotations (∼10°). With the T4 placement, our means are more removed from those obtained with the optoelectronic device (7–20°). Setting aside the fact that the population of the work by Tousignant et al. [27] will produce a result different than our population due to having different demographic characteristics, it seems that measurements taken with the second sensor placed on C7 can be considered more valid.

With that said, none of our results is outside the ROM established in Table 5 and, consequently, it is possible to confirm the validity of the sensor for all CROM motions.

### 4.3. Half vs. Full Movements

The *Sensor Validation Results* section shows that the reliability is higher (better ICC and narrower CI for ICC) when measuring full movements (flexion-extension, lateral extension and rotation) rather than half movements (flexion, extension, right and left lateral flexions, and right and left rotations). We believe that this is due to small discrepancies in the neutral position, which is set by following tips derived from the practitioner’s experience. Since the neutral position calibration error is a problem that all other CROM measurement devices suffer from, using full movements represent an advantage unique to our solution.

The traditional CROM measurements used by practitioners rely on half movements. That said, under many circumstances, total CROM for a given motion is more important than the comparison between the two corresponding half movements. In these cases, measuring full movements (in several or all three axes) does not diminish the value of the data but produces a more reliable result. Our results suggest that, when possible, it is better to rely on full movements, since the variability caused by a drift in the neutral position is eliminated.

### 4.4. Sensor Placement for CROM

In light of the results, an important aspect of the protocol that merits study is the placement of the second sensor, which can be set on the T4 (suggested by the literature [34]) or the C7 vertebrae (suggested by the practitioners we have consulted).

The T4 placement offers only slightly better reliability that C7 in two of the three axes. However, the results for the lateral flexion motion (both full- and half- movements) show better reliability for this particular axis using T4 (see Table 1, Table 2, Table 3 and Table 4).

We also found out that, for the same set of subjects, the ranges with the C7 placement are always lower than those obtained with the second sensor at T4. Concerning validity, both ranges fall within the expected values, but the C7 setup measurements are more similar to the published results using an optoelectronic device [27]. However, this result must be interpreted with caution, since the demographic characteristics of our population is different, and the normal CROM ranges are broad and are heavily influenced by age and sex.

It is also important to note that the subjects reported that the C7 placement was uncomfortable, since the head tends to touch the sensor during extension motions. This is a feasible explanation for the lower ranges measured with this setup.

All factors considered, T4 is, from our point of view, the preferred placement. However, some special conditions or subjects might justify putting the second sensor at C7, since this is the most prominent vertebrae. Such examples include measurements where the lateral flexion motion is less important, or rehabilitation sessions performed without a practitioner (subjects at their own homes, who might find C7 easier to locate).

### 4.5. Future Work

The combination of the solution’s portability and a software component that has been designed for extensibility suggests two areas where promising further developments can happen.

Current rehabilitation methodologies include many monotonous and repetitive exercises, which patients deem uninteresting. Adherence and effectiveness of such treatment can be noticeably improved by using serious videogames, since they offer playful scenarios that make the therapy more attractive [43,44,45]. We are in the process of testing several videogames designed to connect to the described solution, building on its extensible API and real-time sampling of the subject’s movements. On top of that, the portability, easy setup and low cost of the system enables patients to continue their treatment from the comfort of their own homes, with the CROM data being sent to the practitioner automatically through the Internet.

Finally, we have described how the solution measures CROM, since neck pain represents a high prevalence health issue. However, the two-sensor schema is flexible enough to be easily adapted for other joints’ ROM. Currently testing with lumbar, elbow, knee and ankle is undergoing and the results are promising.

## 5. Conclusions

This article describes the development and validation of a new solution for measuring CROM using wearable MEMS-based inertial sensors, a software application and a measurement protocol. This is an inexpensive, small, light and easy to use solution. We have demonstrated its reliability and accuracy by comparing the proposed sensors with optical tracking devices (interrater ICC = 0.93) and carrying out a protocol designed to assess test-retest reliability. Regarding validity, our results fall within the ROM established on previous works.

We defined two protocols (half or full movements), depending on the preferences of the practitioners or the particular circumstances. We also studied two placement configurations for the second sensor (T4 and C7). T4 offers a better test-retest reliability and it is the location usually recommended in the literature. Nevertheless, C7 provides an easier sensor placement, ideal for sessions performed by patients at home or without a practitioner. The proposed protocols fulfill the needs for clinical practice in terms of effectiveness, efficiency and user’s satisfaction.

## Figures and Tables

**Table 1:** Results for the two sets of motions performed with the second sensor located in C7 considering half movements.

	Set 1 (Mean ± SD)	Set 2 (Mean ± SD)	ICC	95% CI for ICC	Mean Difference between Sets	Difference between Means
Flexion	49.54° ± 12.24°	47.82° ± 10.08°	0.87	0.71–0.94	6.10°	1.73°
Extension	47.81° ± 14.14°	47.27° ± 13.24°	0.93	0.84–0.97	5.47°	0.54°
Right Lateral Flexion	33.32° ± 6.51°	34.93° ± 6.75°	0.87	0.70–0.94	3.36°	1.62°
Left Lateral Flexion	35.37° ± 8.18°	35.16° ± 9.71°	0.96	0.90–0.98	2.85°	0.21°
Right Rotation	63.55° ± 10.73°	63.5° ± 9.73°	0.91	0.81–0.96	4.75°	0.06°
Left Rotation	68.64° ± 11.63°	69.47° ± 12.33°	0.91	0.80–0.96	5.27°	0.83°

**Table 2:** Results for the two sets of motions performed with the second sensor located in T4 considering half movements.

	Set 1 (Mean ± SD)	Set 2 (Mean ± SD)	ICC	95% CI for ICC	Mean Difference between Sets	Difference between Means
Flexion	56.92° ± 11.57°	56.14${}^{\circ }$ ± 11.10${}^{\circ }$	0.90	0.77–0.95	5.23°	0.78${}^{\circ }$
Extension	60.53° ± 16.62°	61.25° ± 14.77°	0.92	0.82–0.96	6.65°	0.72${}^{\circ }$
Right Lateral Flexion	38.1° ± 7.26°	37.05° ± 7.69°	0.88	0.74–0.95	3.6°	1.05°
Left Lateral Flexion	38.01° ± 8.29°	40.14° ± 9.00°	0.70	0.36–0.86	5.15°	2.13°
Right Rotation	71.24° ± 10.56°	73.84° ± 11.60°	0.91	0.79–0.96	4.98${}^{\circ }$	2.6${}^{\circ }$
Left Rotation	75.38${}^{\circ }$ ± 10.17${}^{\circ }$	74.2° ± 10.70${}^{\circ }$	0.92	0.82–0.96	4.59${}^{\circ }$	1.18${}^{\circ }$

**Table 3:** Results for the two sets of motions performed with the second sensor located in C7 considering full movements.

	Set 1 (Mean ± SD)	Set 2 (Mean ± SD)	ICC	95% CI for ICC	Mean Difference between Sets	Difference between Means
Flexion-Extension	97.37° ± 21.16°	95.10° ± 20.43°	0.97	0.93–0.99	5.33°	2.27°
Lateral Flexion	68.70° ± 13.18°	70.10° ± 14.49°	0.95	0.89–0.98	4.80°	1.40°
Rotation	132.20° ± 20.43°	132.98° ± 19.20°	0.98	0.95–0.99	4.53°	0.78°

**Table 4:** Results for the two sets of motions performed with the second sensor located in T4 considering full movements.

	Set 1 (Mean ± SD)	Set 2 (Mean ± SD)	ICC	95% CI for ICC	Mean Difference between Sets	Difference between Means
Flexion-Extension	117.46° ± 23.26°	117.40° ± 19.95°	0.97	0.93–0.97	5.14°	0.06°
Lateral Flexion	76.12° ± 14.40°	77.20° ± 13.81°	0.90	0.78–0.95	5.70°	1.08°
Rotation	146.62° ± 17.74°	148.05° ± 20.37°	0.96	0.90–0.98	5.62°	1.43°

**Table 5:** Descriptive statistics for CROM (*n* = 55, healthy subjects, optoelectronic device [27]).

Cervical Movement	Mean ± SD	Range
Flexion	46.1° ± 10.8°	26–77°
Extension	48.8° ± 14.9°	25–83°
Right Lateral Flexion	32.4° ± 8.0°	10–51°
Left Lateral Flexion	33.6° ± 7.3°	10–52°
Right Rotation	55.1° ± 8.9°	25–78°
Left Rotation	57.1° ± 10.3°	28–86°
